# Design, Synthesis, Anticancer Evaluation and Docking Studies of Novel Heterocyclic Derivatives Obtained via Reactions Involving Curcumin

**DOI:** 10.3390/molecules23061398

**Published:** 2018-06-08

**Authors:** Rita M. Borik, Nagwa M. Fawzy, Sherifa M. Abu-Bakr, Magdy S. Aly

**Affiliations:** 1 Department of Chemistry, Jazan University, Jazan 45142, Saudi Arabia; 2 Department of Natural and Microbial Products, National Research Center, Dokki, Cairo 12622, Egypt; fmnagwa@yahoo.com (N.M.F.); sherifakandel@hotmail.com (S.M.A.-B.); 3 Genetics & Cell Biology Division, Department of Zoology, Faculty of Science, Beni-Suef University, Beni-Suef 62511, Egypt; dr.magdy@science.bsu.edu.eg

**Keywords:** furochromone carbaldehyde, Curcumin, anticancer activity, cytotoxicity, human cancer cell lines, molecular docking

## Abstract

Curcumin, a widely utilized flavor and coloring agent in food, has been shown to demonstrate powerful antioxidant, antitumor promoting and anti-inflammatory properties in vitro and in vivo. In the present work, synthesis of new heterocyclic derivatives based on Curcumin was studied. Compound **3** was synthesized via the reaction of furochromone carbaldehyde (**1**) with Curcumin (**2**) using pipredine as catalyst. Also, novel, 4,9-dimethoxy-5*H*-furo [3, 2-*g*] chromen-5-one derivatives **4a**–**d**, **6a**–**d**, **7**, **8a**–**d**, **9** and **10** were synthesized by the reactions of furochromone carbaldehyde (**1**) with different reagents (namely: appropriate amine **3a**–**d**, appropriate hydrazine **5a**–**d**, hydroxylamine hydrochloride, urea/thiourea, malononitrile, malononitrile with hydrazine hydrate). The structure of the synthesized products had been confirmed from their spectroscopic data (IR, ^1^H-NMR, ^13^C-NMR and mass spectra). In the present investigation, the newly synthesized products were screened using the MTT colorimetric assay for their in vitro inhibition capacity in two human cancer cell lines (hepatocellular carcinoma (HEPG2) and breast cancer (MCF-7) as well as the normal cell line (human normal melanocyte, HFB4) in comparison to the known anticancer drugs: 5-flurouracil and doxorubicin. The anticancer activity results indicated that the synthesized products **4c** and **8b** showed growth inhibition activity against HEPG2 cell line and synthesized products **4b** and **8a** showed growth inhibition activity against MCF-7, but with varying intensities in comparison to the known anticancer drugs, 5-flurouracil and doxorubicin. Cyclin dependent kinase 2 (CDK2), a major cell cycle protein, was identified as a potential molecular target of Curcumin. Furthermore, Curcumin induced G1 cell cycle arrest, which is regulated by CDK2 in cancer cells. Therefore, we used molecular modelling to study in silico the possible inhibitory effect of CDK2 by Curcumin derivatives as a possible mechanism of these compounds as anticancer agents. The molecular docking study revealed that compounds **4b**, **8a** and **8b** were the most effective compounds in inhibiting CDk2, and, this result was in agreement with cytotoxicity assay.

## 1. Introduction

Heterocyclic compounds are considered an exceptionally important class of compounds which play a key role in health care and prescription drug design [1]. Currently, several heterocyclic chemical substances are available commercially as anti-cancer drugs.

Curcumin is one of the key components of the curcuminoids group. It is the bioactive ingredient of turmeric. Curcumin is considered an outstanding source of new medication development [2]. Novel studies have shown that curcuminoids have a variety of natural activities, including antioxidant [3,4], anti-inflammatory [5,6], anticancer [7,8] and anti-angiogenesis [9,10] properties, as well as medicinal applications, including their use in HIV therapies [11] and against Alzheimer’s disease [12]. The non-toxic food origin and wide range of pharmaceutical drug properties of curcuminoids ensure they are promising lead molecules for medicinal chemistry. Efforts have taken place to synthesize new curcuminoid derivatives with better biological activities [13,14,15].

On account of these facts, we aimed to prepare novel heterocyclic compounds with different methods from natural products, namely: Curcumin and formyl furochromone. The newly synthesized compounds were screened for their in vitro growth inhibition characterization. The in vitro antiproliferative activity of each compound in the study has been determined using the MTT colorimetric assay [16,17] in hepatocellular carcinoma cell line HEPG2 and breast carcinoma cell line MCF-7 as well as the normal cell line (human normal melanocyte, HFB4).

Computational methods such as molecular docking is very useful and reasonably reliable for prediction of putative binding modes and affinities of ligands for macromolecules. Such methods are gaining popularity because the experimental determination of complex structures is rather difficult and expensive. Over the years, the speed and accuracy of computational docking methods has improved, and these methods now play a significant role in structure-based drug design.

## 2. Results and Discussion

### 2.1. Chemistry

**Scheme 1:**
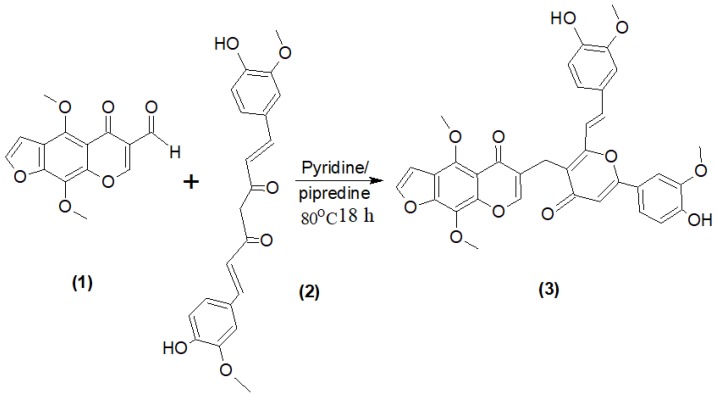
Synthesis of compound **3**.

In an initial study, a typical Knoevenagele Doebner condensation procedure was performed, which involved heating under reflux for a few hours an equimolar quantity of the furochromone carbaldehyde (**1**), Curcumin (**2**) and a base such as pipredine (a few drops) in pyridine as a solvent [18,19]. Compound **3** (Scheme 1) was thus obtained and identified by spectral data such as its IR spectrum, which showed the disappearance of (CHO gp) and the appearance of (OH gp).

**Scheme 2:**
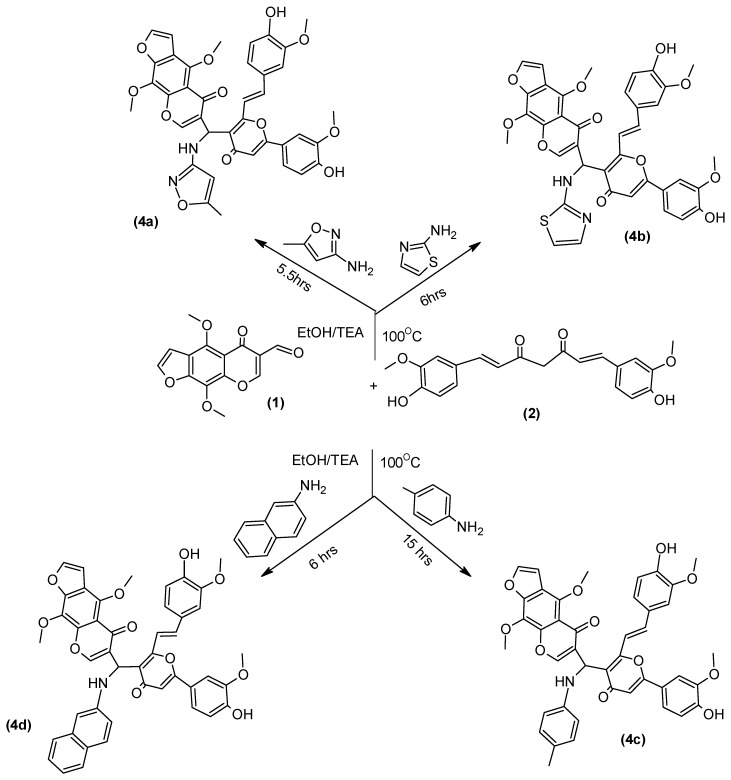
Synthesis of compound **4a**–**d**.

Multicomponent reactions (MCRs) are frequently used in synthetic, medicinal, and combinatorial chemistry [20,21]. DHP derivatives [22,23] are an important class of heterocycles due to their biological pharmacological activities. The reaction conditions were optimized after attempting reactions of equimolar amounts of the furochromone carbaldehyde (**1**) Curcumin (**2**) and various amines **3a**–**d**, namely: 3-amino-5-methylisoxazole, thiazol-2-amine, *p*-toluidine and 1-naphthylamine respectively, in presence of absolute alcohol/TEA. Formation of compounds **4a**–**d** (Scheme 2) was monitored by TLC using ethyl acetate: petroleum as eluent.

**Scheme 3:**
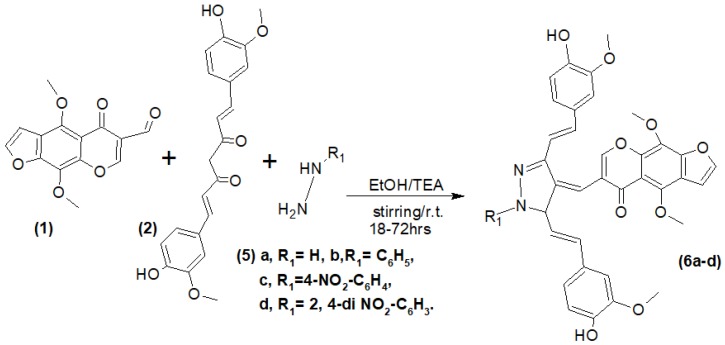
Synthesis of compounds (**6a**–**d**).

Heterocyclic compounds have significant importance due to their therapeutic properties [24]. Also, heterocycles containing pyrazole scaffolds [25,26] exhibit some interesting biological activities. Herein, the heterocyclic compounds **6a**–**d** containing pyrazole rings (Scheme 3) were obtained *via* the condensation reaction of hydrazine derivatives **5a**–**d** (namely: hydrazine hydrate, phenyl hydrazine 4-nitrophenyl hydrazine and 2,4-nitrophenyl hydrazine) with furochromone carbaldehyde (**1**) and Curcumin (**2**) in the presence of ethanol/TEA for an appropriate time.

**Scheme 4:**
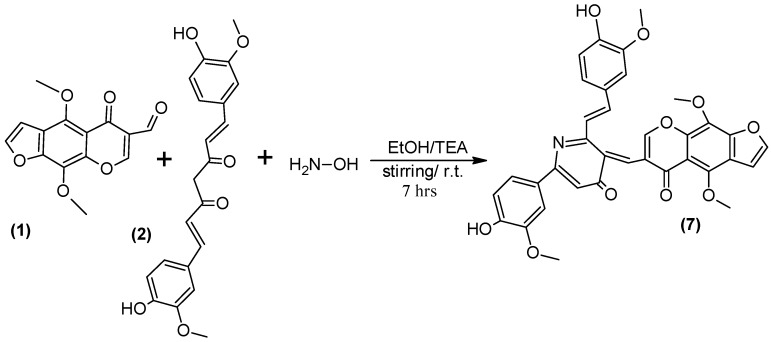
Synthesis of compound (**7**).

We also report herein a one-pot protocol for the synthesis of compound **7** (Scheme 4) by a three-component condensation of furochromone carbaldehyde (**1**), Curcumin (**2**) and hydroxylamine hydrochloride in ethanol using TEA as catalyst.

**Scheme 5:**
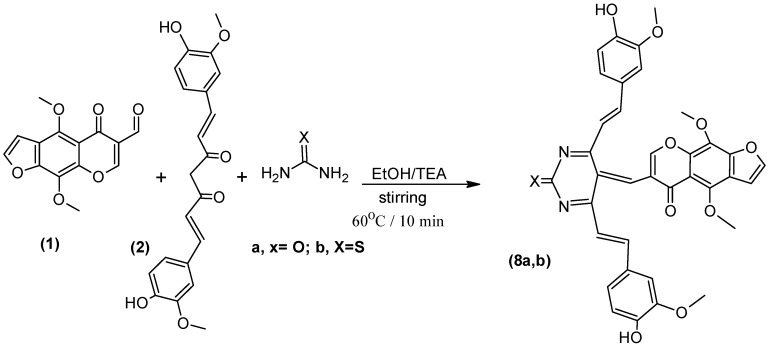
Synthesis of compound (**8a**,**b**).

The Biginelli reaction [27,28,29] is an important MCR [30] because the products exhibit a wide range of biological activities. An acid-catalyzed Biginelli reaction involving a one-pot three component condensation of furochromone carbaldehyde (**1**), Curcumin (**2**), and urea/thiourea gave compounds **8a**,**b** (Scheme 5).

**Scheme 6:**
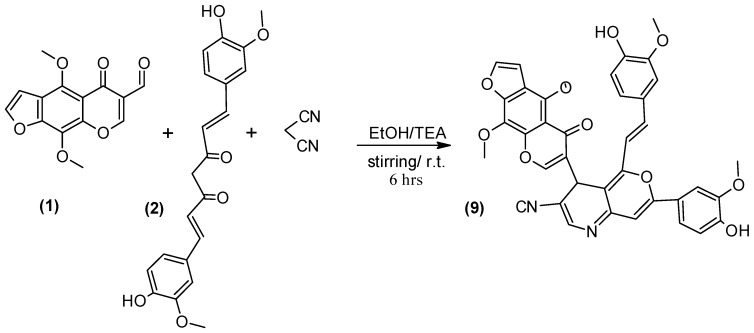
Synthesis of compound (**9**).

Nitrogen heterocycles [31] are very important class of chemicals for the synthesis of novel drugs. Here, we report a procedure for the one-pot combination of furochromone carbaldehyde (**1**), Curcumin (**2**), and malononitrile [32] to synthesis compound **9** (Scheme 6).

**Scheme 7:**
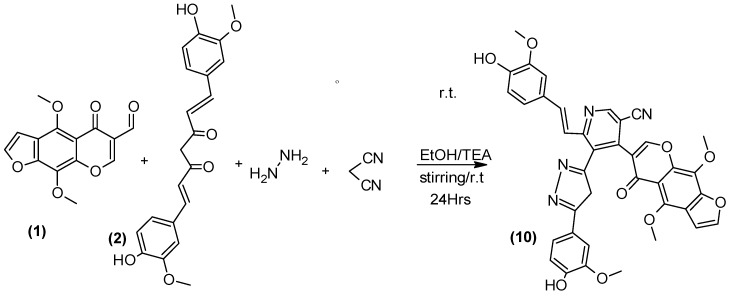
Synthesis of compound **10**.

Derivatives of *o*-aminocarbonitrile [33] are considered important organic intermediates because they have many applications in the synthesis of heterocyclic compounds. One pot MCRs [34] were utilized to prepare the target compound **10** (Scheme 7) by the reaction of furochromone carbaldehyde (**1**), Curcumin (**2**), hydrazine hydrate and malononitrile.

All the synthesized compounds were characterized using IR, ^1^H-NMR, ^13^C-NMR and mass spectrometry.

### 2.2. Anticancer Activity

Most of the anti-tumor drugs currently used in chemotherapy are toxic to normal cells and cause toxicity for immune cells, so it is important to minimize doses to the least amount possible as well as try to minimize the side effects of these drugs. Therefore, the identification of new anti-cancer drug with low side effects on the immune system has become an essential goal in many immuno-pharmacology studies [35].

The newly synthesized products were evaluated for their in vitro cytotoxic activity against human hepatocellular carcinoma (HEPG2) and breast carcinoma (MCF-7) cell lines. Doxorubicin (DOX) and 5-fluorouracil (5-FU), which are two of the most effective anticancer agents, were used as reference drugs.

Our results showed that some of the newly synthesized products exhibited a moderate to strong growth inhibition activity on the tested cell lines between 0 and 50 μg/mL concentrations in comparison to the reference anticancer drugs. The relationship between surviving fraction and drug concentration was plotted to obtain the survival curve of the two cell lines. The response parameter calculated was the IC_50_ value, which corresponds to the concentration required for 50% inhibition of cell viability. Table 1 shows the in vitro cytotoxic activity of the synthesized compounds, where some compounds exhibited significant activity compared to the reference drugs.

Some of the tested compounds showed remarkable anticancer activity against MCF-7 cancer cells, while compounds **3**, **4a**, **4c**, **4d**, **6a**, **6c**, **6d**, **7**, **8b**, **9** and **10** had no effect on the breast cells. In the same sense, evaluation the anticancer effect of the tested compounds against human liver HEPG2 cancer cell line revealed that although compounds **3**, **4a**, **4b**, **4d**, **6a**, **6c**, **6d**, **7**, **8a**, **9** and **10** had no antiproliferative effect, compounds **3c** and **8b** showed anticancer activity close to that of the standard drug.

**Figure 1:**
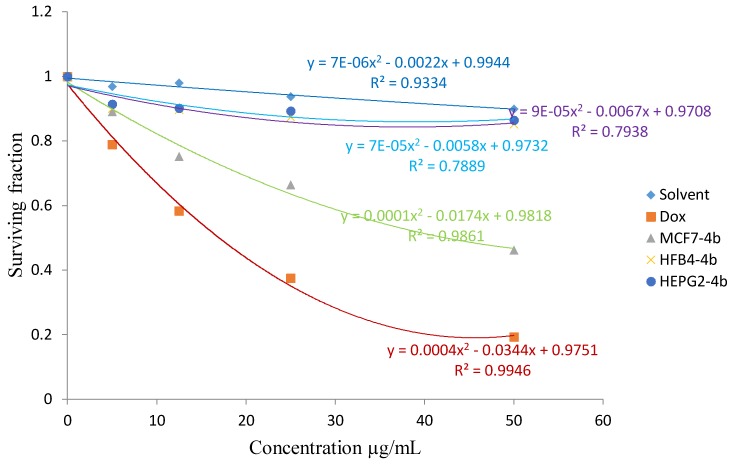
The cytotoxic activity of the synthesized product **4b** against breast MCF7 cancer cell line.

**Figure 2:**
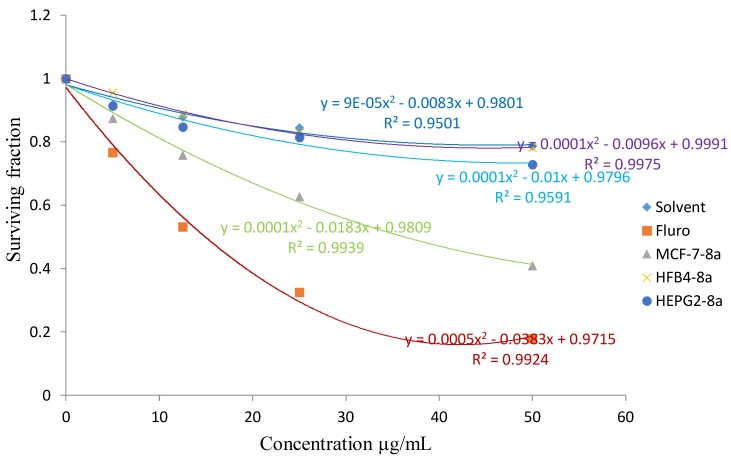
The cytotoxic activity of the synthesized product **8a** against breast MCF7 cancer cell line.

**Figure 3:**
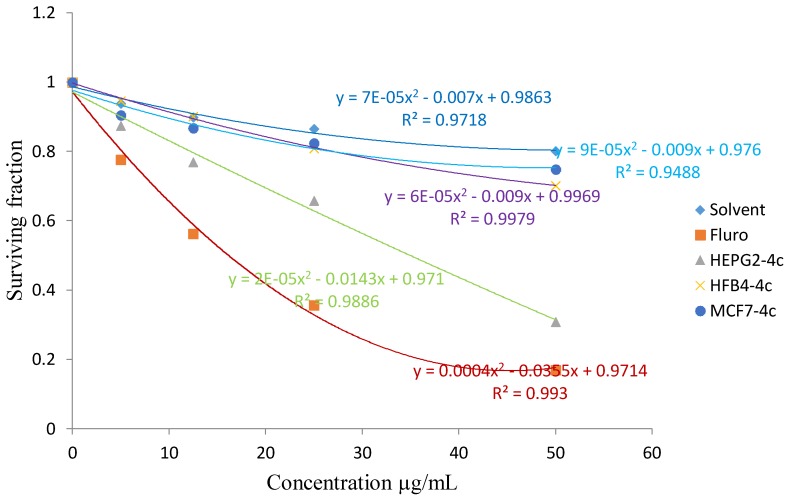
The cytotoxic activity of the synthesized product **4c** against liver HEPG2 cancer cell line.

**Figure 4:**
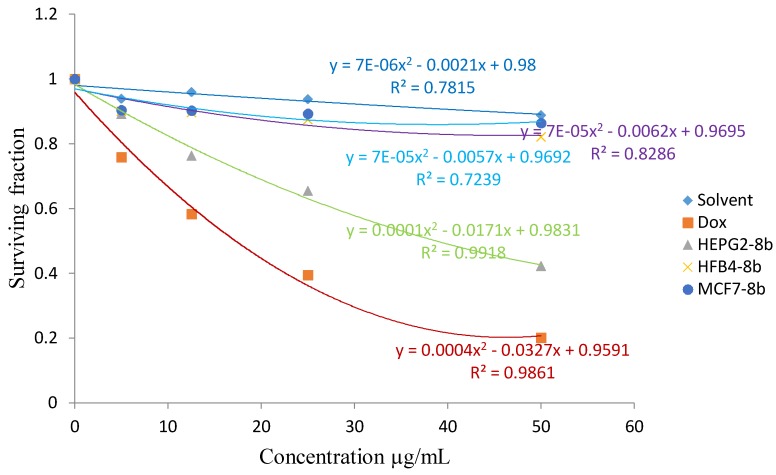
The cytotoxic activity of the synthesized product **8b** against liver HEPG2 cancer cell line.

Figure 1 shows the cytotoxic activity of the synthesized product **4b** and Figure 2 shows the cytotoxic activity of the synthesized product **8a**, against breast MCF7 cancer cell line. Figure 3 shows the activity of the compound **4c**, and Figure 4 shows the activity of the compound **8b** against HEPG2 cell line in comparison to the reference drugs 5-FU and DOX.

From the results listed in Table 1, compounds **4b** and **8a** showed in vitro cytotoxic activity with IC_50_ values of 20 and 23, respectively, for the MCF-7 cell line. Compounds **4c** and **8b** showed an in vitro cytotoxic activity with IC_50_ values of 18 and 23, respectively for the HEPG2 cell line when the cells were subjected to different concentrations of the compounds.

Additionally, the results revealed that most of the compounds exhibited no activity against the growth of normal HFB4 cells.

### 2.3. Molecular Docking Results

To ensure that the binding poses of the docked compounds represented favorable and valid potential binding modes, the docking parameters and methods were validated by redocking the cocrystal ligand in order to determine the ability of Auto Dock vina to reproduce the orientation and position of the ligand observed in the crystal structure. The redocking of cocrystal ligands to their respective molecular targets exhibited an RMSD value of <2 Å between the original cocrystal ligand position and the docked poses, which was RMSD = 1.047 Å. This confirmed that the ligands were closely bound to the true conformation of their targets indicating the reliability of the docking protocols and parameters.

**Figure 5:**
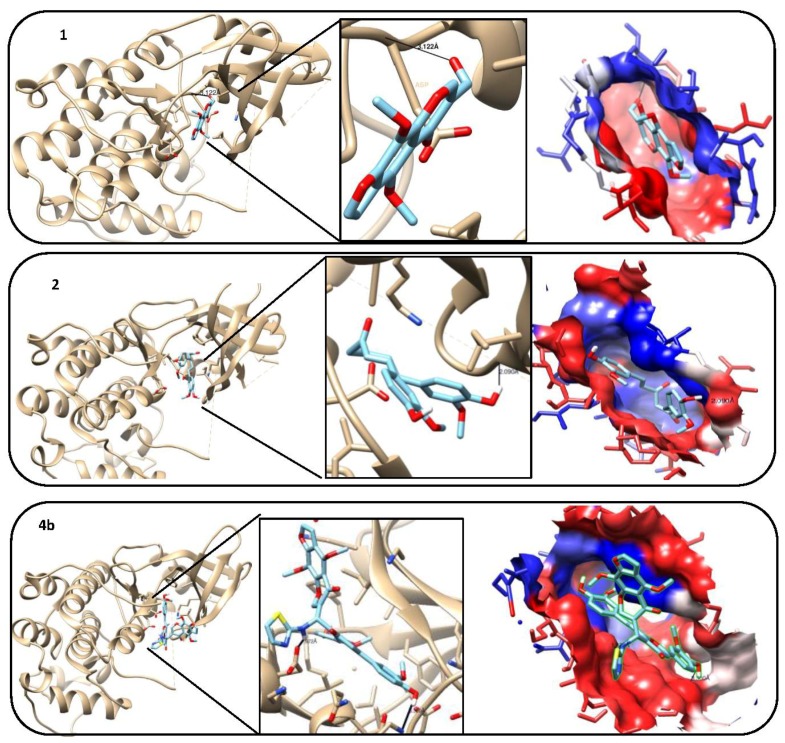

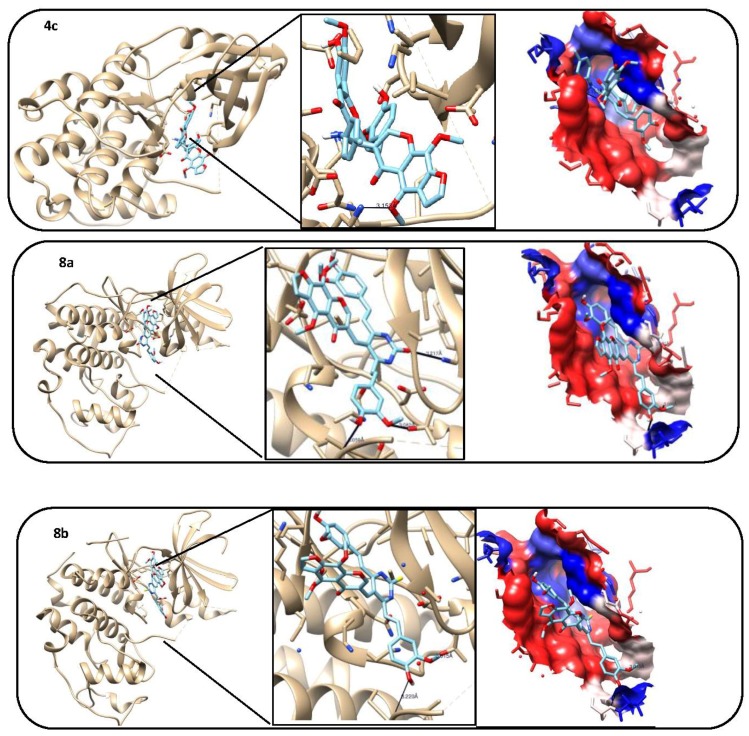
3D model, hydrogen bond formation, and hydrophobic interaction between tested compounds (**1**, **2**, **4b**, **4c**, **8a** and **8b**) and cyclin dependent Kinase 2 (CDK2) protein.

The molecular docking studies revealed that the compounds **4b**, **8a** and **8b** were the most promising compounds, which is explained by lowest binding energy, hydrogen bonding and hydrophobic interactions with the active site residues of CDK2 and that might be one of the reasons for the good activities shown by these compounds in in vitro studies (Table 2 and Table 3), and Figure 5.

## 3. Experimental Section

### 3.1. General Information

All melting points were uncorrected and were taken on electro-thermal capillary melting point apparatus. The melting points were measured in degrees centigrade and determined using a Büchi 510 apparatus (Hi Tech trager, NRC, Cairo, Egypt). IR spectra were recorded on a Mattson-5000 FTIR spectrometer (LabX, Midland, ON, Canada) using the KBr wafer technique. ^1^H-NMR, ^13^C-NMR spectra were determined on a Varian-Gemini-300 MHz (Facility of pharmacy, Cairo university, Egypt) or Jeol Ex-300 MHz ^1^NMR spectrometer (NRC, Cairo, Egypt) using TMS (with chemical shift δ = 0 ppm) as an internal standard. Mass spectra were determined on a Finnigan MatSSQ 7000 instrument in EI mode (70 EV) (Thermo Inst. Sys. Inc., Waltham, MA, USA). All reactions were monitored by TLC using pre-coated plastic sheet silica gel (0.25 mm, 20 × 20 cm, 60F254, E. Merck KGaA, Konstanz, Germany) and spots were visualized by irradiation with UV light (254 nm). 

*6-((2-(4-Hydroxy-3-methoxystyryl)-6-(4-hydroxy-3-methoxyphenyl)-4-oxo-4H-pyran-3-yl)methyl)-4,9-dimethoxy-5H-furo[3,2-g]chromen-5-one* (**3**): A mixture of furochromone carbaldehyde (**1**, 1 mmol), Curcumin (**2**, 1 mmol), was refluxed at 80 °C in a round bottomed flask containing pyridine (10 mL) and pipredine (a few drops) as catalyst. The reaction mixture was then heated at 80 °C for 18 h (till completion of the reaction checked by TLC (pet. ether: ethyl acetate 1:2). After completion of the reaction, the mixture was cooled to room temperature and poured into ice and acidified. The crude product **3** was obtained by simple filtration and purified by recrystallization from ethanol. Yellow color powder, m.p. 178 °C, 66% yield. Mol. formula: C_35_H_28_O_11_, Mol. Wt.: 624.59. Elemental analysis: calc: C, 67.30; H, 4.52, found: C, 66.87; H, 4.57. IR (KBr, cm^−1^) bands at 1650, 1787 (2C=O), and br. 3432 (2OH). ^1^H-NMR (DMSO-*d*_6_, δ, ppm): 2.35 (s, 2H, CH_2_), 3.88 (s, 12H, 4OCH_3_); 5.43, (s, 2H, OH phenol ring, exchangeable D_2_O), 6.11, 6.22 (d, 2H, *J* = 2.00, CH=CH), 6.54–6.76 (m, 7H, arom. + Pyran ring), 6.56, 7.86 (dd, 2H, *J* = 2.01, furan ring); 7.77 (s, 1H, H_7_). ^13^C-NMR (100 MHz, DMSO-*d*_6_): d 25.0, 39.32, 39.52, 39.73, 39.94, 60.91, 66.11, 66.12, 72.03, 72.40, 73.65, 73,77, 79.13, 92.11, 96.4, 102.5, 106.0, 111.8, 112, 112.6, 112.9, 115.3, 116.8, 117.1, 120.1, 120.9, 124.0, 124.5, 135.2, 146.0, 147.3, 149.7, 177.0, 179.21, 180.8. Exact Mass: 626.18. Exact Mass: 624.16, m/e: 624.11 (44.5%), 623.10 (24.3%), 622.11 (18.3%).92.21,

*General procedure to prepare compounds***4a**–**d**: A mixture of furochromone carbaldehyde (**1**, 1 mmol), Curcumin (**2**, 1 mmol) and an amine derivative (namely: 3-amino-5-methylisoxazole, thiazol-2-amine, 1-naphthylamine or *p*-toluidine) was refluxed at 100 °C in absolute alcohol (10 mL) using TEA as a catalyst for 6 h–18 h. The reaction was monitored via TLC (pet. ether: ethyl acetate 1:2). Then the solvent was removed via rotatory evaporation and the product was obtained by filtration and recrystallization from suitable solvent.

*6-((2-(4-Hydroxy-3-methoxystyryl)-6-(4-hydroxy-3-methoxyphenyl)-4-oxo-4H-pyran-3-yl)(5-methyl-isoxazol-3-ylamino)methyl)-4,9-dimethoxy-5H-furo[3,2-g]chromen-5-one* (**4a**): Yellow color powder , m.p. 156 °C decomposition, yield 55%, recrystallization of ethyl acetate and few drops of methanol. Mol. formula: C_39_H_32_N_2_O_12_, Mol. Wt.: 720.68. Elemental analysis: calc: C, 65.00; H, 4.48; N, 3.89, found C, 64.68 H, 4.01; N, 3.33. IR (KBr, cm^−1^) bands at 1634, 1776 (2C=O), and br. 3435 (2OH). ^1^H-NMR (DMSO-*d*_6_, δ, ppm): 1.18 (s, 3H, CH_3_), 3.40, 3.51 (s, 12H, 4OCH_3_); 2,66, 5,47 (dd, 2H, NH-CH, exchangeable for NH D_2_O), 4.58 (s, 1H, isoxazole ring), 4.60, 4.61 (s, 2H, OH phenol ring, exchangeable D_2_O), 5.47 (s, 1H, pyran ring), 5.49, 5.50 (d, 2H, *J* = 2.11, CH=CH), 6.54–6.66 (m, 6H, arom), 6.56, 7.66 (dd, 2H, *J* = 2.01, furan ring); 8.10 (s, 1H, H_7_). Exact Mass: 720.2, m/e: 719.9 (10.0%), 718.20 (49.1%), 717.11 (41.4%).

*6-((2-(4-Hydroxy-3-methoxystyryl)-6-(4-hydroxy-3-methoxyphenyl)-4-oxo-4H-pyran-3-yl)(thiazol-2-ylamino)methyl)-4,9-dimethoxy-5H-furo[3,2-g]chromen-5-one* (**4b**): Brown color powder, m.p. 152 °C decomposition, 35% yield, recrystallization from chloroform. Mol. formula: C_38_H_30_N_2_O_11_S, Mol. Wt.: 722.72. Elemental analysis: calc: C, 63.15; H, 4.18; N, 3.88; S, 4.44, found: C, 62.68; H, 3.86; N, 3.35; S, 4.11. IR (KBr, cm^−1^) bands at 1666, 1732 (2C=O), and br. 3334 (OH). ^1^H-NMR (DMSO-*d*_6_, δ, ppm): 3.89 (s, 12H, 4OCH_3_), 3.99 (s, 1H, CH), 4.21, (dd, 2H, NH-CH, exchangeable D_2_O), 6.44 (d, 1H, CH pyran ring), 6.34, 6.72 (dd, 2H, *J* = 2.01, CH=CH), 6.51, 7.62 (dd, 2H, 2H isothiazol ring), 5.43, (d, 2H, OH phenol ring, exchangeable D_2_O), 6.74–6.86 (m, 6H, arom), 6.56, 7.66 (dd, 2H, *J* = 2.01, furan ring); 8.00 (s, 1H, H_7_). ^13^C-NMR (100 MHz, DMSO-*d*_6_): d 39.12, 39.34, 39.65, 39.99, 56.2, 56.3, 60.82, 72.7, 73.57, 79.18, 95.1, 96.4, 102.5, 106.0, 111.8, 112, 112.6, 112.9, 115.3, 116.8, 117.1, 120.1, 120.9, 124.0, 124.5, 135.2, 144.8, 146.0, 147.3, 149.7, 150.0, 150.8, 151.3, 153.6, Exact Mass: 722.16, m/e: 722.16 (100.0%), 722.16 (42.7%), 721.14 (11.3%), 720.15 (4.5%).

*6-((2-(4-Hydroxy-3-methoxystyryl)-6-(4-hydroxy-3-methoxyphenyl)-4-oxo-4H-pyran-3-yl)(p-toluidino)-methyl)-4,9-dimethoxy-5H-furo[3,2-g]chromen-5-one* (**4c**): Yellow color powder, m.p. 250 °C decomposition, 36% yield, recrystallization from chloroform. Mol. formula: C_42_H_35_NO_11_, Mol. Wt.: 729.73. Elemental analysis: calc: C, 69.13; H, 4.83; N, 1.92, found: C, 68.78; H, 4.42; N, 1.78. IR (KBr, cm^−1^) bands at 1665, 1754 (2 C=O), and br.3443 (2OH). ^1^H-NMR (DMSO-*d*_6_, δ, ppm): 2.55 (s, 2H, CH_2_), 3.86 (s, 12H, 4OCH_3_); 4.18, (dd, 2H, NH-CH, exchangeable D_2_O), 4.74 (s, 1H, pyran ring), 5.00 (d, 2H, OH phenol ring, exchangeable D_2_O), 6.63, 6.81 (d, 2H, *J* = 2.11, CH=CH), 6.34–6.76 (m, 10H, arom), 6.56, 7.66 (dd, 2H, *J* = 2.01, furan ring); 8.00 (s, 1H, H_7_). Exact Mass: 729.22, m/e: 729.20 (100.0%), 728.22 (45.8%), 727.11 (12.7%).

*6-((2-(4-Hydroxy-3-methoxystyryl)-6-(4-hydroxy-3-methoxyphenyl)-4-oxo-4H-pyran-3-yl)(naphthalen-2-ylamino)methyl)-4,9-dimethoxy-5H-furo[3,2-g]chromen-5-one* (**4d**): Yellow color powder, m.p. 234 °C decomposition, 47% yield, Mol. formula: C_45_H_35_NO_11_, Mol. Wt.: 765.76. Elemental analysis: calc: C, 70.58; H, 4.61; N, 1.83, found: C, 70.02; H, 4.21; N, 1.11. IR (KBr, cm^−1^) bands at 1661, 1762 (2C=O), and br.3333 (OH). ^1^H-NMR (DMSO-*d*_6_, δ, ppm): 2.26 (s, 2H, CH_2_), 3.95 (s, 12H, 4OCH_3_); 4.14, (dd, 2H, NH-CH, exchangeable D_2_O); 5.65, (s, 1H, pyran ring), 5.13, (d, 2H, OH phenol ring, exchangeable D_2_O), 6.32, 6.22 (d, 2H, *J* = 2.00, CH=CH), 6.33–6.65 (m, 13H, arom), 6.56, 7.66 (dd, 2H, *J* = 2.01, furan ring); 7.76 (s, 1H, H_7_). Exact Mass: 765.22, m/e: 765.00 (10.0%), 764.02 (49.0%), 763.23 (14.2%).

*General procedure to prepared compounds***6a**–**d**: A mixture of furochromone carbaldehyde (**1**, 1 mmol), curcumin (**2**, 1 mmol) and a hydrazine derivative (namely: hydrazine hydrate, phenyl hydrazine 4-nitrophenyl hydrazine or 2,4-dinitrophenyl hydrazine) was stirred in ethanol(20mL)/TEA(2drops) at room temperature and appropriate time from 18 to 72 h. The progress of the reaction was monitored by TLC using ethyl acetate: petroleum ether (30:70, *v*/*v*) as eluent. After completion of the reaction, water (5 mL) was added to the reaction mixture. The solid separated was collected by filtration at the pump and further purified by recrystallization from an appropriate solvent.

*6-((3,5-bis(4-Hydroxy-3-methoxystyryl)-4H-pyrazol-4-ylidene)methyl)-4,9-dimethoxy-5H-furo[3,2-g]-chromen-5-one* (**6a**): Orange color powder, m.p. 240 °C decomposition, 45% yield, recrystallized from ethanol. Mol. formula: C_35_H_28_N_2_O_9_, Mol. Wt.: 620.6. Elemental analysis: calc: C, 67.74; H, 4.55; N, 4.51, found: C, 67.33; H, 4.32; N, 4.00. IR (KBr, cm^−1^) bands at 1625 (C=O), and br. 3333 (OH) and 3254 (NH). ^1^H-NMR (DMSO-*d*_6_, δ, ppm): 3.88 (s, 12H, 4OCH_3_), 4.11 (s, 1H, pyrazole ring), 5.22, (s, 2H, OH, exchangeable D_2_O), 6.22 (s, 1H, CH), 6.64, 6.43 (dd, 4H, *J* = 2.22, 2CH=CH), 6.44–6.66 (m, 6H, arom), 6.73, 7.93 (dd, 2H, *J* = 2.01, furan ring); 8.02 (s, 1H, H_7_). ^13^C-NMR (100 MHz, DMSO-*d*_6_): d 39.12, 39.32, 39.73, 39.91, 40.25, 40.38, 60.99, 72.03, 72.70, 73.655, 79.13, 101.00, 116.8, 117.1, 120.1, 120.9, 124.0, 124.5, 135.2, 144.6, 144.9, 146.0, 144.9, 146.0, 147.3, 149.7, 150.8, 151.3, 151.6, 153.6, 155.6, 157.1, 177.5. Exact Mass: 620.18, m/e: 620.11 (40.0%), 619.12 (33.0%), 618.19 (11.8%).

*6-((3,5-bis(4-Hydroxy-3-methoxystyryl)-1-phenyl-1H-pyrazol-4(5H)-ylidene)methyl)-4,9-dimethoxy-5H-furo[3,2-g]chromen-5-one* (**6b**): Yellow color powder, m.p. 236 °C decomposition, 48% yield, recrystallized from chloroform. Mol. formula: C_41_H_34_N_2_O_9_. Mol. Wt.: 698.72. Elemental analysis: calc: C, 70.48; H, 4.90; N, 4.01, found: C, 69.67; H, 4.53; N, 3.87. IR (KBr, cm^−1^) bands at 1643 (C=O), and br.3363 (OH). ^1^H-NMR (DMSO-*d*_6_, δ, ppm): 3.77 (s, 12H, 4OCH_3_), 4.34 (s, 1H, pyrazole ring), 5.13, (s, 2H, OH, exchangeable D_2_O), 6.34 (s, 1H, CH), 6.65, 6.48 (dd, 4H, *J* = 2.24, 4CH=CH), 6.31–6.70 (m, 11H, arom), 6. 65, 7.66 (dd, 2H, *J* = 2.01, furan ring); 7.65 (s, 1H, H_7_). Exact Mass: 698.23, m/e: 698.20(10.0%), 697.22 (45.1%), 696.11 (11.9%).

*6-((1Z)-(3,5-bis(4-Hydroxy-3-methoxystyryl)-1-(4-nitrophenyl)-1H-pyrazol-4(5H)-ylidene)methyl)-4,9-dimethoxy-5H-furo[3,2-g]chromen-5-one* (**6c**): Yellow Color, m.p. 233 °C, 88% yield, recrystallized from chloroform, Mol. formula: C_41_H_33_N_3_O_11_, Mol. Wt.: 743.71. Elemental analysis: calc: C, 66.21; H, 4.47; N, 5.65, found: C, 66.01; H, 4.20; N, 5.33. IR (KBr, cm^−1^) bands at 1630 (C=O), and br. 3423 (OH). ^1^H-NMR (DMSO-*d*_6_, δ, ppm): 3.97 (s, 12H, 4OCH_3_), 4.33 (s, 1H, pyrazole ring), 5.15, (s, 2H, OH, exchangeable D_2_O), 6.31 (s, 1H, CH), 6.43, 6.23 (dd, 2H, *J* = 2.22, 2 CH=CH), 6.61–6.74 (m, 10H, arom), 6.55, 7.21 (dd, 2H, *J* = 2.01, furan ring); 7.98 (s, 1H, H_7_). Exact Mass: 743.21, m/e: 743.21 (53.0%), 742.01 (41.5%), 741.10 (12.0%).

*6-(3,5-bis(4-Hydroxy-3-methoxystyryl)-1-(2,4-dinitrophenyl)-1H-pyrazol-4(5H)-ylidene)methyl)-4,9-dimethoxy-5H-furo[3,2-g]chromen-5-one* (**6d**): Orange color powder, m.p. 244 °C, 90% yield, recrystallized from chloroform, Mol. formula: C_41_H_32_N_4_O_13_. Mol. Wt.: 770.72. Elemental analysis: calc: C, 63.89; H, 4.18; N, 9.09, found C, 63.56; H, 3.76; N, 8.78. IR (KBr, cm^−1^) bands at 16621 (C=O), and br. 3443 (OH). ^1^H-NMR (DMSO-*d*_6_, δ, ppm): 3.87 (s, 12H, 4OCH_3_), 4.42 (s, 1H, pyrazole ring), 5.32, (s, 2H, OH, exchangeable D_2_O), 6.00 (s, 1H, CH), 6.32, 6.54 (dd, 4H, *J* = 2.20, CH=CH), 6.67–6.76 (m, 9H, arom), 6. 65, 7.31 (dd, 2H, *J* = 2.01, furan ring); 7.65 (s, 1H, H_7_). Exact Mass: 770.21, m/e: 770.00 (230%), 699.12 (46.6%), 698 (9.9%).

*Preparation of 2-(4-hydroxy-3-methoxystyryl)-6-(4-hydroxy-3-methoxyphenyl)-3-((4,9-dimethoxy-5-oxo-5H-furo[3,2-g]chromen-6-yl)methylene)pyridin-4(3H)-one* (**7**): A mixture of furochromone carbaldehyde (**1**, 1 mmol), Curcumin **2** (1 mmol) and hydroxylamine hydrochloride(1 mmol) was stirred in ethanol(15mL)/TEA(2 drops) for 7 h. at room temperature. The progress of the reaction was monitored by TLC using ethyl acetate: petroleum ether (30:70, *v*/*v*) as eluent. After completion of the reaction, water (5 mL) was added to the reaction mixture. The solid separated was collected by filtration at the pump and further purified by recrystallization from acetonitrile–water mixture (2:1). Yellow color powder, m.p. 252 °C, 78% yield, Mol. formula: C_35_H_27_NO_10_, Mol. Wt.: 621.59. Elemental analysis: C, 67.63; H, 4.38; N, 2.25, found: C, 67.11; H, 4.03; N, 2.00. IR (KBr, cm^−1^) bands at 1660, 1783 (C=O) and 3432 (OH). ^1^H-NMR (DMSO-*d*_6_, δ, ppm): 3.88 (s, 12H, 4OCH_3_); 5.05, (s, 3H, OH, exchangeable D_2_O), 5.35 (s, H, CH), 6.21, 6.51 (dd, 4H, *J* = 2.20, CH=CH), 6.34–6.76 (m, 6H, arom), 6.56, 7.66 (dd, 2H, *J* = 2.01, furan ring). Exact Mass: 621.16, m/e: 621.16 (23.0%), 620.17 (34.6%), 619.17 (13%).

*General procedure to prepare compounds***8a**, **b**: One mmol of urea/or thiourea, respectively, was dissolved in absolute ethanol (30 mL) and this solution was stirred at 60 °C for 10 min. Then curcumin (**2**, 1 mmol) was added to this solution. The reaction mixture was cooled in ice. Furochromone carbaldehyde (**1,** 1 mmol), was added to this mixture and 4 drops of conc. HCl (aq.) were added. After stirring for 7 h the reaction mixture was allowed to warm to room temperature and stirred for an additional 8 h. After removal of the volatiles in vacuo the residue was washed with cold H_2_O (315 mL) and neutralization with NaOH solution affording the products **8a**, **b**.

*4,6-bis(4-Hydroxy-3-methoxystyryl)-5-((4,9-dimethoxy-5-oxo-5H-furo[3,2-g]chromen-6-yl)methylene)pyrimidin-2(5H)-one* (**8a**): Brown color powder, m.p. 274 °C decomposition, 78% yield, recrystallization from EtOH and few drops of acetone. Mol. formula: C_36_H_28_N_2_O_10_, Mol. Wt.: 648.61. Elemental analysis: calc: C, 66.66; H, 4.35; N, 4.32, found: C, 66.23; H, 4.11; N, 4.00. IR (KBr, cm^−1^) bands at 1660, 1721 (2C=O), and 3338 (OH). ^1^H-NMR (DMSO-*d*_6_, δ, ppm): 3.89 (s, 12H, 4OCH_3_); 5.07 (s, 2H, OH, exchangeable D_2_O), 5.33 (s, H, CH), 5.43, 6.55 (dd, 4H, *J* = 2.21, 2CH=CH), 6.34–6.76 (m, 6H, arom), 6.46, 7.67 (dd, 2H, *J* = 2.01, furan ring); 7.78 (s, 1H, H_7_). Exact Mass: 648.17, m/e: 648.11 (11.0%), 647.11 (33.2%), 646.10 (19.0%).

*6-((4,6-bis(4-Hydroxy-3-methoxystyryl)-2-thioxopyrimidin-5(2H)-ylidene)methyl)-4,9-dimethoxy-5H-furo[3,2-g]chromen-5-one* (**8b**): Brown color powder, m.p. 254 °C decomposition, 88% yield, recrystallization from ethyl acetoacetate. Mol. formula: C_36_H_28_N_2_O_9_S, Mol. Wt.: 664.68. Elemental analysis: calc: C, 65.05; H, 4.25; N, 4.21 S, 4.82, found: C, 64.78; H, 3.98; N, 4.01; S, 4.50. IR (KBr, cm^−1^) bands at 1645 (C=O), and 3398 = (OH). ^1^H-NMR (DMSO-*d*_6_, δ, ppm): 3.88 (s, 12H, 4OCH_3_); 5.05, (s, 2H, OH, exchangeable D_2_O), 5.33 (s, H, CH), 5.41, 5.49 (dd, 4H, *J* = 2.21, 2CH=CH), 6.51–6.66 (m, 6H, arom), 6.56, 7.66 (dd, 2H, *J* = 2.01, furan ring); 7.70 (s, 1H, H_7_). ^13^C-NMR (100 MHz, DMSO-*d*_6_): d 39.32, 39.52, 39.73, 39.94, 56.40, 76, 60.91, 72.03, 72.40, 73.65, 74.3, 79.13, 88.7, 100.00, 111.8, 112.0, 112.9, 116.8, 120.1, 121.00, 124.0, 124.5, 144.9, 146.0, 147.3, 150.8, 151.3, 153.6, 154.9, 156, 157.1, 164.6, 171.5, 177.5, 220.0. Exact Mass: 664.15, m/e: 664.10 (18.9%), 663.10 (50.5%), 662.10 (22.4%).

*5-(4-Hydroxy-3-methoxystyryl)-7-(4-hydroxy-3-methoxyphenyl)-4-(4,9-dimethoxy-5-oxo-5H-furo[3,2-g]chromen-6-yl)-4H-pyrano[4,3-b]pyridine-3-carbonitrile* (**9**): A mixture of furochromone carbaldehyde (**1**, 1 mmol), Curcumin (**2**, 1 mmol) and malononitrile (1 mmol) was stirred in ethanol (10 mL) and TEA as a catalyst for 6 h at room temperature (the progress of the reaction was monitored by TLC). After completion, the reaction mixture was filtered, and the precipitate was crystallized by chloroform/ethanol. Yellow color powder, m.p. 220 °C decomposition, 76% yield, Mol. formula: C_38_H_28_N_2_O_10_, Mol. Wt.: 672.64. Elemental analysis: calc: C, 67.85; H, 4.20; N, 4.16; found: C, 67.51; H, 3.99; N, 3.87; IR (KBr, cm^−1^) bands at 1650 (C=O) and 2220 (CN). ^1^H-NMR (DMSO-*d*_6_, δ, ppm): 3.88 (s, 12H, 4OCH_3_); 5.44, (s, 2H, OH, exchangeable D_2_O), 5.32, 6.54 (dd, 2H, *J* = 2.20, CH=CH), 6.34–6.76 (m, 6H, arom), 6.56, 7.66 (dd, 2H, *J* = 2.01, furan ring); 7.70 (s, 1H, H_7_), 8.34 (s, H, H pyridine ring. Exact Mass: 672.17, m/e: 672.00 (20.3%), 671.18 (43.8%), 670.18 (21.3%).

*6-(4-Hydroxy-3-methoxystyryl)-5-(5-(4-hydroxy-3-methoxyphenyl)-4H-pyrazol-3-yl)-4-(4,9-dimethoxy-5-oxo-5H-furo[3,2-g]chromen-6-yl)pyridine-3-carbonitrile* (**10**): A mixture of furochromone carbaldehyde (**1**, 1 mmol), Curcumin (**2**, 1 mmol), hydrazine hydrate (1 mmol) and malononitrile (1 mmol) was stirred in ethanol (10 mL) and TEA as a catalyst for 24 h at room temperature (The progress of the reaction was monitored by TLC). After completion, the reaction mixture was filtered, and the precipitate was crystallized by chloroform. Yellow color powder, m.p. 240 °C, 87% yield. Mol. formula: C_38_H_28_N_4_O_9_, Mol. Wt.: 684.65. Elemental analysis: calc: C, 66.66; H, 4.12; N, 8.18; found: C, 66.11; H, 3.67; N, 7.88; IR (KBr, cm^−1^) bands at 1662 (C=O), 2224 (CN) and 3441 (OH). ^1^H-NMR (DMSO-*d*_6_, δ, ppm): 1.46 (s, 2H, CH_2_, pyrazole ring), 3.88 (s, 12H, 4OCH_3_); 5.05, (s, 2H, OH, exchangeable D_2_O), 6.34–6.76 (m, 6H, arom), 6.56, 7.66 (dd, 2H, *J* = 2.01, furan ring), 7.70 (s, 1H, H_7_) and 9.70 (s, 1H, H pyridine ring). Exact Mass: 684.19, m/e: 684.11 (10.0%), 683.19 (33.4%), 682.19 (19.9%).

### 3.2. Determination of Anticancer Activities

#### 3.2.1. Cell Culture

For anticancer activity screening of the tested compounds, liver HepG2 and breast MCF-7, cell lines as well as the normal cell line (human normal melanocyte, HFB4) were obtained from the National Cancer Institute, Cairo University, Cairo, Egypt. The cells were maintained in Dulbecco’s modified Eagle’s medium (DMEM) supplemented with 10% heat inactivated fetal calf serum (Hyclone, Waltham, MA, USA), penicillin (100 U/mL) and streptomycin (100 μg/mL) at 37 °C in humidified atmosphere containing 5% CO_2_. Cells at a concentration of 0.50 × 10^6^ were grown in a 25 cm^2^ flask in 5 mL of culture medium.

#### 3.2.2. In vitro Cell Proliferation and Cell Viability Assay—Trypan Blue Exclusion Assay

Trypan blue exclusion assay was performed as previously described [36]. This assay is used to assess the effect of newly synthesized products on viability of HEPG2 and MCF7 cells. Approximately 0.75 × 10^5^ cells/mL was seeded in a six well tissue culture plate and different concentrations of compounds were added after 24 h. For the determination of growth rate, smaller aliquots were collected in a 0.5 mL tubes, trypan blue (0.4%) was added to the cell suspension, and the number of cells viable cells (unlabeled), number of non-viable cells (blue), and the number of damaged cells (slightly blue) was determined using a haemocytometer. Viability is just the ratio of live cells divided by total number of cells. The media was not changed during the induction period. Each experiment was repeated a minimum of three times and the results are presented as graphs.

#### 3.2.3. MTT Assay

The synthesized products were subjected to a screening system for evaluation of their anticancer activity against hepatocellular carcinoma HEPG2 cell line and breast carcinoma MCF-7 cell line in comparison to the known anticancer drugs: 5-FU and DOX. Cell survival was further assessed by the 3-(4,5-dimethylthiazol-2-yl)-2,5-diphenyl tetrazolium bromide (MTT) dye reduction assay which is based on the ability of viable cells to metabolize a yellow tetrazolium salt to violet form azan product that can be detected spectrophotometrically. Exponentially growing cells (HEPG2 and MCF-7) were plated in triplicate in 96-well sterilized plates at a density of 1 × 10^4^ cells/well. After 24 h, cells were treated with escalating doses of the compound under investigation and incubated in 5% CO_2_ atmosphere with high humidity. After 48 and 72 h of compound exposure, the cells were incubated with MTT (0.5 mg/mL) for another 4 h at 37 °C. The blue MTT formazan precipitate was then, solubilized in detergent (50% final concentration of *N*,*N*-dimethylformamide and 10% of sodium dodecyl sulphate) and incubated for an additional 2 h. Absorbance was measured at 570 nm on a multi-well ELISA plate reader. The mean absorbance of medium control was the blank and was subtracted. IC50 values (concentration of compound causing 50% inhibition of cell growth) were estimated after 72 h exposure of compound. The absorbance of control cells was taken as 100% viability and the values of treated cells were calculated as a percentage of control. The 5-fluorouracil and doxorubicin anticancer drugs were used as positive control, and cells without samples were used as negative control. The relation between surviving fraction and drug concentration is plotted to get the survival curve of both cancer cell lines with the specified compound. A statistical significance was tested between samples and negative control (cells with vehicle) using independent t-test by the SPSS 11 program (SPSS Inc., Chicago, IL, USA). DMSO is the vehicle used for dissolution of products.

### 3.3. Molecular Docking Study

The structures of all tested compounds were modeled using the Chemsketch software (http://www.acdlabs.com/resources/freeware/). The structures were optimized and energy minimized using the VEGAZZ software [37]. The optimized compounds were used to perform molecular docking. The 3D structure of the molecular target was obtained from Protein Data Bank (PDB) (www.rcsb.org): CDK-2 (PDB: 1di8, https://www.rcsb.org/pdb/explore/explore.do?structureId=1di8). The steps for receptor preparation included the removal of heteroatoms (water and ions), the addition of polar hydrogen, and the assignment of charges. The active sites were defined using grid boxes of appropriate sizes around the bound cocrystal ligands, which was like this: number of grid points (60 × 60 × 60), center ((xyz coordinates)—7.623, 49.88, 11.36), and the grid point spacing 0.375 Å. The tested compounds were docked into the active site of the CDK2, to study their interaction in silico and to correlate their anti-cancer activity. The docking study was performed using Auto dock vina [38], and Chimera for visualization [39].

## 4. Conclusions

In conclusion, we have reported the multi-component reaction of furochromone carbaldehyde (**1**) and curcumin (**2**), with different amines, hydrazines, urea/thiourea, malononitrile and malononitrile with hydrazine hydrate in a simple one-pot for the synthesis of a series of new 5*H*-furo[3,2-*g*]chromene derivatives **4a**–**d**, **6a**–**d**, **8**–**10**. The newly synthesized compounds were screened for their in vitro inhibition capacity in two human cancer cell lines HEPG2 and MCF-7 using 5-flurouracil and doxorubicin as reference drugs. Some of newly synthesized products exhibited a moderate to good growth inhibition activity, where compound **8b** showed the highest activity against HEPG-2 cells, and compound **4b** showed the highest activity against MCF7 cells. The molecular docking study revealed that compounds **4b**, **8a** and **8b** were the most effective compounds in inhibiting CDk2, and, these results were in agreement with the cytotoxicity assay results. 

## Figures and Tables

**Table 1:** IC_50_ of the newly synthesized products against the two cell lines.

Compound	Cell Lines	
	MCF-7	HEPG-2
Solvent	75.78	75.78
**1**	53	50
**3**	33	28
**4a**	33	26
**4b**	20	50
**4c**	33	18
**4d**	37	22
**6a** (N5007)	26	29
**6c** (N50011)	38	42
**6d**	40	42
**7**	28	35
**8a**	23	49
**8b**	53	23
**9**	29	36
**10**	37	44
5-Flurouracil	13.35	
Doxorubicin		14.70

**Table 2:** The results of molecular docking of best conformer with 1di8 receptor.

Compound	Binding Energy (Kcal/mol)	No. of H-Bonds	Length of H-Bonds	Formed Amino Acids with H-Bonds
Reference ligand (DTQ: 4-[3-Hydroxyanilino]-6,7-Dimethoxyquinazoline)	−8.3	2	3.162 Å	LEU83A
			3.245 Å	LYS33A
**1**	−7.4	1	3.122 Å	ASP145A
**2**	−8.9	1	2.090 Å	GLU12A
**4b**	−8.8	2	2.750 Å	LYS 33A
			1.921 Å	GLN 131A
**4c**	−8.4	1	3.153 Å	LYS 89A
**8a**	−9.4	3	3.217 Å	LYS 33A
			3.059 Å	THR165A
			3.082 Å	THR14A
**8b**	−9.3	2	3.220 Å	THR165A
			3.015 Å	THR14A

**Table 3:** The hydrophobic interactions of best conformer with 1di8 receptor.

Compound	Hydrophobic Interactions
Reference ligand (DTQ: 4-[3-Hydroxyanilino]-6,7-Dimethoxyquinazoline)	ILE10, VAL18, LEU148, VAL64, LEU134, LEU83
**1**	ILE10, VAL18, LEU134, VAL64, LEU83, PHE82
**2**	ILE10, VAL18, LEU83, LEU134, LEU148, VAL64,
**4b**	ILE10, VAL18, LEU148, VAL64, LEU134, LEU83, LEU298, PHE80
**4c**	ILE10, VAL18, LEU134, VAL64, LEU83, LEU298
**8a**	ILE10, VAL18, VAL64, VAL164, LEU298, LEU134, LEU83, PHE80
**8b**	ILE10, VAL18, VAL64, VAL164, LEU298, LEU134, LEU83, PHE80
